# Peptic ulcer disease among dyspeptic patients at endoscopy unit, University of Gondar hospital, Northwest Ethiopia

**DOI:** 10.1186/s12876-022-02245-6

**Published:** 2022-04-05

**Authors:** Belete Assefa, Abilo Tadesse, Zenahebezu Abay, Alula Abebe, Tsebaot Tesfaye, Melaku Tadesse, Ayenew Molla

**Affiliations:** 1 Department of Internal Medicine, School of Medicine, College of Medicine and Health Sciences, University of Gondar, Gondar, Ethiopia; 2 Department of Epidemiology and Biostatistics, Institute of Public Health, College of Medicine and Health Sciences, University of Gondar, Gondar, Ethiopia

**Keywords:** Dyspepsia, Peptic ulcer disease, *H. pylori*, NSAIDs, Northwest Ethiopia

## Abstract

**Background:**

Dyspepsia is a common complaint in upper gastrointestinal disorders. It is described as predominant epigastric pain lasting for at least one month. Globally, peptic ulcer disease occurs in 3.5–32% of patients with dyspepsia. *Helicobacter pylori* (*H. pylori*) infection and non-steroidal anti-inflammatory drugs/aspirin use are the widely known risk factors for peptic ulcer disease. There was no recent document on *H. pylori* infection rate among patients with peptic ulcer disease in Ethiopia. This study aimed to determine magnitude and associated factors of peptic ulcer disease among dyspeptic patients in Northwest Ethiopia.

**Methods:**

An institutional-based cross sectional study was conducted at the University of Gondar hospital, Northwest Ethiopia. A convenience sampling method was used to recruit 218 study subjects. A pre-designed semi-structured questionnaire was used to extract clinical information. Olympus flexible fiber-optic endoscope (Olympus, GIF-E 600, Olympus Corp., Hamburg, Germany) was used to confirm the presence of peptic ulcer disease. Diagnosis of active *H. pylori* infection was made using the fecal *H. pylori* Antigen 25 T Card Test (Anamol Lab., Pvt. Ltd., Palghar, India). The Data were entered into EPI Info version 4.6.0.2, and then exported to SPSS version 20 for analysis. Explanatory variables associated with peptic ulcer disease were analyzed by applying logistic regression model. *P* value < 0.05 was used to declare significant association.

**Result:**

A total of 218 dyspeptic patients who underwent upper gastrointestinal endoscopic evaluations were included in the study. The mean (+ SD) age of patients was 42 ± 16.4 years. Forty nine percent (95% CI 42.4–56.2) of dyspeptic patients had active *H. pylori* infection. Peptic ulcer disease was diagnosed in 35% (95% CI 31.4–39.2) of patients with dyspepsia. *H. pylori* infection (AOR = 6.298, 95% CI 2.965–13.378, *P* value <  0.001) and NSAIDs/ASA use (AOR = 6.252, 95% CI 2.925–13.362, *P* value <  0.001) were identified as risk factors for peptic ulcer disease.

**Conclusion:**

Medical treatment of peptic ulcer disease should target treatment of *H. pylori* infection and cautious use of non-steroidal anti-inflammatory drugs/aspirin.

## Background

Dyspepsia is a common complaint in upper gastrointestinal disorders. It is described as predominant epigastric pain lasting for at least one month [1, 2]. It can manifest as postprandial fullness, early satiety, or epigastric burning or pain. Globally, dyspepsia occurs in 10–20% of adults, and accounts for 3% of medical office visits. Dyspepsia has an impact on the quality of life of patients and the expenses to the health care service [1, 2]. According to previous studies, peptic ulcer disease (PUD) occurred in 2.4–3.5% of the Western populace, 12–15% of Asian inhabitants, and 24–28% of sub-Saharan African dwellers [3–8]. *Helicobacter pylori* (*H. pylori*) infection and non-steroidal anti-inflammatory drugs (NSAIDs)/aspirin (ASA) use are the major culprits for causing gastroduodenal mucosal injuries [9–14]. *H. pylori* is widely known to cause gastritis and peptic ulcer disease. Also, *H. pylori* is attributed to gastric cancer and gastric B-cell lymphoma, and is categorized as a class I carcinogen by the International Agency for Research on Cancer (IARC), a division of the World Health Organization (WHO) [9–12]. Use of NSAIDs is recognized to cause erosive gastritis and peptic ulcer disease. Prevalence of PUD was documented to be 14–25% among NSAIDs/ASA users [13–15]. Other less frequently implicated risk factors for PUD include genetics, stress, diet, smoking and alcohol [16, 17]. Global reports indicated that *H. pylori* infection rate among PUD patients was 14–21% in the United States, 60–70% in Asia, and 70–90 in sub-Saharan Africa [4, 5, 7, 18–23]. There was no recent document on *H. pylori* infection rate among PUD patients in Ethiopia. There are several invasive and non-invasive diagnostic tests to detect *H. pylori* infection. Invasive tests include endoscopic biopsy specimen for histology, culture and rapid urease test (RUT) and polymerase chain reaction (PCR). Non-invasive tests consist of urea breath test (UBT), serum antibody test, stool antigen test, saliva antibody test and urinary antibody test [24, 25]. The choice of diagnostic tests is based on the prevalence of *H. pylori* infection, the availability and cost of the diagnostic tests, and patient-related characteristics [19–23]. This study used fecal *H. pylori* antigen test to document active *H. pylori* infection, which has optimal diagnostic accuracy. The study aimed to determine the magnitude, *H. pylori* infection rate, and associated factors of PUD among dyspeptic patients at the University of Gondar hospital, Northwest Ethiopia. The study would give valuable information on the approach to treating dyspepsia in the setting and similar institutions.

## Methods

### Study design and setting

An institutional-based cross sectional study was conducted at the endoscopy unit, University of Gondar hospital, between June 1, 2020 and November 30, 2020. The hospital is located in Northwest Ethiopia, which is 750 km away from the capital, Addis Ababa. The hospital had a catchment population of 5 million people. The endoscopy unit was established in the hospital in 2000. It has provided endoscopic services for patients with gastrointestinal disorders. It was staffed by trained internists and surgeons, unit nurses, and a cleaner. The upper gastrointestinal endoscopy sessions were done three days per week, and on average, five to eight patients attended each of endoscopic days. The other two days of a week were for colonoscopy sessions.

### Study population

All patients who underwent endoscopic evaluation at endoscopy unit, University of Gondar hospital was the study population.

### Inclusion criteria

Adults 18 years or older who presented with a complaint of dyspepsia, and underwent endoscopic evaluation at the endoscopic unit, University of Gondar during the study period were included in the study.

### Exclusion criteria

Study subjects who were on antibiotics or PPI in the last one month, had alarming gastro duodenal features, had active bleeding diathesis, and didn’t give consent to undergo endoscopic evaluation were excluded from the study.

### Study variables

#### Dependent variable: peptic ulcer disease

Independent variables: (1) socio-demographic characteristics include age, gender, residence, marital status, and socioeconomic status. (2) clinical characteristics include *H. pylori* infection, NSAIDs/ASA use, and presence of co-morbidities such as cardiovascular diseases, rheumatologic diseases, chronic airway diseases, and HIV infection, (3) behavioral factors include cigarette smoking and alcohol consumption.

### Sample size and sampling procedure

The sample size was calculated using a single population proportion formula with the assumption of 95% confidence level, 5% margin of error, and taking a 25% estimated proportion of peptic ulcer disease among dyspeptic patients [7, 8]. The sample size was determined for a study population size of 1000 during the study period. A convenience sampling method was used to recruit 224 study subjects.

### Data collection instrument and procedures

All relevant clinical information, endoscopic findings and *H. pylori* test results were recorded on predesigned semi-structured questionnaire.

### Clinical procedures

Patients were interviewed to obtain socio-demographic data and relevant clinical history before the upper gastrointestinal endoscopy. The clinical history includes indication for endoscopy (dyspepsia), duration of dyspepsia, use of NSAIDs/ASA, history of cigarette smoking and alcohol consumption, and presence of co-morbidities. Focused physical examination was done to each of the patients.

### Endoscopic procedures

All endoscopic procedures were conducted by trained physicians (internists and surgeons). Olympus flexible fiber-optic endoscope (Olympus GIF-E 600, Olympus Corp., Hamburg, Germany) was used for the procedure. Informed consent was obtained from all patients before the procedure. Lidocaine (2%) throat spray and IV midazolam (2 mg/ml) were used as local anesthetic and sedative agents respectively. All procedures were conducted in the morning on an empty stomach. Diagnoses of endoscopic appearances (site, size, and number of gastro duodenal lesions) were at the discretion of the endoscopist.

### Diagnosis of *H. pylori* infection

Diagnosis of active *H. pylori* infection was made using the ‘Fecal *H. pylori* Antigen 25 T Card Test’ (Anamol Lab., Pvt. Ltd., Palghar, India). Fecal *H. pylori* antigen test is a lateral flow chromatographic immunoassay for qualitative detection of *H. pylori* antigen in human fecal specimen. Positive test result indicates an active *H. pylori* infection.

### ‘Fecal *H. pylori* Antigen 25 T Card Test’ procedures

The membrane strip was inserted into the cassette. Adequate volume of fecal specimen was dispensed into the sample well of the test cassette. The *H. pylori* antigen in the stool sample (for positive test) reacted with the *H. pylori* antibody conjugate in the test device. The immune-complex moved along the membrane chromatographically to the test region. The pre-coated antibody of the test-band captured the immune-complex to give the test result. Positive *H. pylori* test was indicated by the red color test-band. Negative test result showed no color change in test-band. Internal procedural control was included in the test. A red colored band appearing in the control region was the internal procedural control. The test result was interpreted within 15 min of the procedure.

### Data analysis

Data were entered into and cleaned in EPI Info™ version 4.6.0.2 (EPI Info™ Inc., Atlanta, USA) and transported to and analyzed in SPSS version 20 (SPSS Inc., Chicago, USA). Categorical variables were reported as frequencies (percentages) and continuous variables as mean with standard deviation. The results were summarized by using frequency, tables and graphs. Risk factors for PUD were analyzed by applying logistic regression model. The goodness of fit of the model was judged from the Hosmer–Lemeshow test. The fit of the model was considered acceptable (*P* value = 0.67). Those variables with a *P* value < 0.25 in the bi-variate analysis were exported to multi-variate analysis. The results were presented as odds ratio with 95% confidence interval. *P* value < 0.05 was used to declare significant association.

### Ethical considerations

The research protocol complied with the Declaration of Helsinki and ethical clearance was obtained from the Institutional Review Board (IRB) of the College of Medicine and Health Sciences, University of Gondar (19/02/2020, IRB No. 1267/02/2020). Study subjects were recruited only after written informed consent was obtained. All data obtained were treated confidentially. Dyspeptic patients who were found to have endoscopic proven peptic ulcer disease and positive fecal *H. pylori* antigen test were taken care of as per the recommendation of 2017 ACG clinical guideline: Treatment of *Helicobacter pylori* infection [26].

### Definition of terms

Dyspepsia is predominant epigastric pain lasting for at least one month [1].

Peptic ulcer disease is a visible defect in the gastric or duodenal mucosa more than 5 mm with peripheral edema and overlying white exudate [9].

Alarming gastro duodenal features include family history of gastrointestinal cancer, intractable vomiting, progressive dysphagia or odynophagia, anemia, unexplained weight loss, or gastrointestinal bleeding (hematemesis or melena).

NSAIDs/ASA user refers to a patient who consumes NSAIDs/ASA at any dosage for at least three months [27].

Cigarette smoker refers to someone who has smoked more than 100 cigarettes in their lifetime and has smoked in the last 28 days [28].

Alcohol consumption is defined as alcohol intake more than two drinks for men and one drink for women in a day [29].

## Results

### Socio-demographic characteristics of study subjects

Two hundred eighteen patients were included in the study to give a response rate of 97.3%. Six (2.7%) study subjects were exempted from statistical analysis due to incomplete data. The mean (+ SD) age of the patients was 42 ± 16.4 years. Majority of study subjects were males (118/218, 54%), urban residents (126/218, 58%) married (139/218, 64%), and had joined school (146/218, 67%). Most (186/218, 85%) of study subjects were Orthodox Christian followers (Table 1). More than a third (78/218, 36%) had a history of alcohol consumption, while less than five percent (10/218, 4.6%) were cigarette smokers (Table 2).

**Table 1:** Socio-demographic characteristics of dyspeptic patients, who underwent upper gastrointestinal endoscopic evaluation at endoscopy unit, University of Gondar hospital, June1, 2020 to November 30, 2020 (n = 218)

Characteristics	Category	Frequency	Percentage
Age	18–28	59	27.1
	29–40	56	25.7
	41–55	51	23.4
	56+	52	23.8
Sex	Male	118	54.1
	Female	100	45.9
Marital status	Single	58	26.6
	Married	139	63.8
	Divorced	14	6.4
	Widowed	7	3.2
Residence	Urban	126	57.8
	Rural	92	42.2
Religion	Orthodox Christian	186	85.2
	Protestant Christian	7	3.2
	Muslim	25	11.6
Level of education	Didn’t join school	72	33.1
	Elementary school	44	20.2
	Secondary school	40	18.3
	College graduate	31	14.2
	Degree graduate and above	31	14.2

**Table 2:** Clinical and behavioral characteristics of dyspeptic patients, who underwent upper gastrointestinal endoscopic evaluation at endoscopy unit, University of Gondar hospital, June1, 2020 to November 30, 2020 (n = 218)

Characteristics	Frequency (No.)	Percentage (%)
*H. pylori* infection		
Yes	107	49.1
No	111	50.9
NSAIDs/ASA use		
Yes	86	39.5
No	132	60.5
Co-morbidities		
Yes	43	19.7
No	175	80.3
Cigarette smoking		
Yes	10	4.6
No	208	95.4
Alcohol consumption		
Yes	78	35.8
No	140	64.2

### Clinical characteristics of study subjects

#### Clinical features

The average duration of dyspepsia was 12.4 (± 7.6) months, which ranged from 2 months to 4 years. Abdominal tenderness was elicited in one-third (63/218, 29%) of patients.

#### Endoscopic findings

**Fig. 1:**
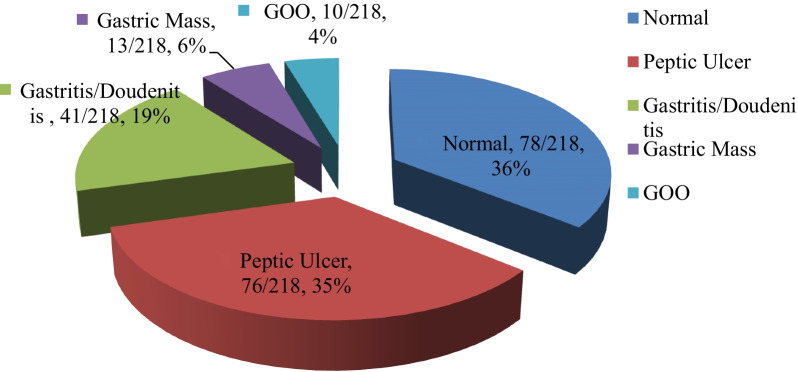
Endoscopic findings among patients with dyspepsia

Peptic ulcer disease (PUD) was diagnosed in 35% (95% CI 31.4–39.2) of patients with dyspepsia. Two-thirds (55/76, 72%) of PUD cases had duodenal ulcers. Other organic causes of dyspepsia were gastritis/doudenitis (41/218, 19%), gastric mass (13/218, 6%) and pyloric obstruction (10/218, 4%). More than one-third (78/218, 36%) had functional dyspepsia (Fig. 1).

#### *H. pylori* infection rate

Forty nine percent (95% CI 42.4–56.2), 107/218, of dyspeptic patients had active *H. pylori* infection (Table 2). Seventy-one (95% CI 66.7–77.5) 54/76, PUD patients had active *H. pylori* infection. The majority (46/54, 85%) of *H. pylori* infections among PUD patients had duodenal ulcer.

#### NSAIDs/ASA users

NSAIDs/ASA was used by forty percent (86/218, 40%) of dyspeptic patients (Table 2). More than half (47/86, 55%) of NSAIDs/ASA users were diagnosed to have PUD.

#### Co-morbidities

One-fifth (43/218, 20%) of study subjects had co-morbidities, including cardiovascular diseases (25/43, 58%), rheumatologic diseases (12/43, 28%), chronic airway diseases (4/43, 9%), and HIV infection (2/43, 5%).

### Factors associated with risk of developing PUD among dyspeptic patients

Multivariate logistic regression analysis revealed that dyspeptic patients with *H. pylori* infection (AOR = 6.298, 95% CI 2.965–13.378, *P* value = 0.000) and NSAIDs/ASA use (AOR = 6.252, 95% CI 2.925–13.362, *P* value = 0.000) were at risk of developing PUD, while non-married study subjects were protected from developing PUD (AOR = 0.367, 95% CI = 0.154–0.887, *P* value = 0.024) (Table 3). There was no statistically significant difference in the magnitude of peptic ulcer disease among age groups, gender, residence, cigarette smoking, alcohol consumption, and presence of co-morbidities.

**Table 3:** Bi-variable and multi-variable regression analysis of factors associated with peptic ulcer disease in upper gastrointestinal endoscopy evaluated dyspeptic patients at endoscopy unit, University of Gondar hospital, Northwest Ethiopia, June1, 2020 to November 30, 2020 (n-218)

Variables	PUD		COR (CI)	*P* value	AOR (CI)	*P* value
	Yes	No				
Marital status						
Non-married	20	59	0.502 (0.273–0.925)	0.027	0.367 (0.154–0.887)	0.024
Married	56	83	1		1	
Cigarette smoking						
Yes	6	4	2.957 (0.808–10.823)	0.101	3.153 (0.585–16.998)	0.182
No	70	138	1		1	
*H. pylori* infection						
Positive	54	53	4.122 (2.259–7.519)	0.000	6.298 (2.965–13.378)	< 0.001
Negative	22	89	1		1	
NSAIDs/ASA use						
Yes	47	39	4.280 (2.369–7.734)	0.000	6.252 (2.925–13.362)	< 0.001
No	29	103	1		1	

## Discussion

Among a total of 218 dyspeptic patients, active *H. pylori* infection was documented in 49% (95% CI 42.4–56.2) of study subjects. Likewise, the *H. pylori* infection rate among PUD patients was 71% (95% CI 66.7–77.5). These findings were congruent with hospital-based sub-Saharan African (SSA) reports. The African reports verified that 40–65% of dyspeptic and 65–90% PUD patients were positive for *H. pylori* infection [7, 8, 19, 20, 23, 24]. The Ethiopian pooled prevalence of *H. pylori* infection was 52.2% (95% CI 45.8–58.6) in a recent hospital-based meta-analysis [30]. The global magnitude of *H. pylori* infection was 34% in Western Europe, 37% in Northern America, 55% in Asia, and 70% in Africa [31]. The global difference in the magnitude of the *H. pylori* infection rate could be explained by the difference in socio-economic status, environmental sanitation, living conditions, and personal hygiene. In this study, PUD (35%) was the commonly observed abnormal endoscopic lesion, followed by gastritis/duodenitis (19%) and gastric mass (6%). More than one-third (36%) had functional dyspepsia. Recent Nigerian study reported that gastritis/duodenitis (27%) and PUD (28%) were the frequently documented abnormal endoscopic findings. Gastric cancer (2.3%) was less frequently reported [7]. Studies in Tanzania and Ethiopia reported that gastritis/duodenitis (80–98%) followed by PUD (25–32%) were the commonly observed endoscopic pathologies. Gastric cancer was detected in 3–7% of dyspeptic patients [8, 19]. PUD (62%) followed by gastric cancer (12%) was the frequently detected endoscopic finding in Ghanaian study [23]. While, studies in Nigeria and Kenya witnessed gastritis/duodenitis (72–79%) was the commonest endoscopic finding. PUD (6.5–13%) and gastric cancer (1.4–2.3%) were less frequently reported [20, 24]. The difference in the type of gastro duodenal lesions among dyspeptic patients in African reports could be explained by differences in patient-related characteristics (age, genetics), *H. pylori* virulence strain, NSAIDs/ASA exposure rate, lifestyle preferences (smoking, alcohol), and other environmental factors [19, 20, 23, 24]. This study revealed that nearly forty percent (39.5%) of dyspeptic patients had a history of NSAIDs/ASA use, and more than half (55%) of NSAIDs/ASA users developed PUD. Western literature reviews documented that dyspepsia occurred in up to half (50–60%) of patients taking NSAIDs/ASA and up to a third (15–30%) of patients using NSAIDs/ASA developed PUD [13–15]. On multivariable logistic regression analysis, odds of developing PUD was sixfold higher among dyspeptic patients with *H. pylori* infections than those with negative *H. pylori* infections (AOR = 6.298, 95% CI 2.965–13.378, *P* value <  0.001). It was confirmed that *H. pylori* establishes prolonged gastro duodenal mucosal infection, and leads to chronic active gastritis and PUD [9–12, 19, 20, 23, 24]. Dyspeptic patients who use NSAIDs/ASA had sixfold increased risk of developing PUD as compared to non-NSAIDs/ASA users (AOR = 6.252, 95% CI 2.925–13.362, *P* value  <  0.001). NSAIDs/ASA interferes with the cyclo-oxygenase (COX) pathway and depletes biosynthesis of gastric prostaglandins. In addition, NSAIDs/ASA are weak acids which cause direct gastric mucosal toxic injury [9, 10, 13–17]. Study subjects with non-married status were 60% protected from developing PUD as compared to their counter parts (AOR = 0.367, 95% CI = 0.154–0.887, *P* value = 0.024). Reduced family size and non-crowded living condition among non-married subjects might contribute to reduced *H. pylori* infection rate and occurrence of PUD.

## Limitation of the study

The study subjects were referred patients to the hospital, which was more likely to be the severely ill study candidates. In addition, the convenience sampling method might introduce selection bias.

## Conclusions

*H. pylori* infection was often detected in dyspeptic patients. Majority of PUD patients were diagnosed to have *H. pylori* infection. Dyspeptic patients with *H. pylori* infection and NSAIDs/ASA use were at risk of developing PUD.

## Recommendations

Medical treatment of PUD should target treatment of *H. pylori* infection and cautious use of NSAIDs/ASA. Community-based study is required to conclude the actual findings in the target population.

## Data Availability

All data generated and analyzed were included in this research article.

## Abbreviations

ACG: American College of Gasrtoenterology
ASA: Aspirin
AOR: Adjusted odds ratio
CI: Confidence interval
CDC: Center for Disease Control and Prevention
COR: Crude odds ratio
*H. pylori*: *Helicobacter pylori*
IV: Intravenous
NSAIDs: Nonsteroidal anti-inflammatory drugs
PPI: Proton pump inhibitors
PUD: Peptic ulcer disease
